# Improved cold tolerance in switchgrass by a novel CCCH-type zinc finger transcription factor gene, *PvC3H72*, associated with ICE1–CBF–COR regulon and ABA-responsive genes

**DOI:** 10.1186/s13068-019-1564-y

**Published:** 2019-09-20

**Authors:** Zheni Xie, Wenjing Lin, Guohui Yu, Qiang Cheng, Bin Xu, Bingru Huang

**Affiliations:** 1 College of Agro-grassland Science, Nanjing Agricultural University, Nanjing, 210095 People’s Republic of China; 2 Jiangsu Key Laboratory for Poplar Germplasm Enhancement and Variety Improvement, Nanjing Forestry University, Nanjing, 210037 People’s Republic of China; 3 Department of Plant Biology and Pathology, Rutgers the State University of New Jersey, New Brunswick, NJ 08901 USA

**Keywords:** CCCH, Bioenergy, Switchgrass, Chilling, Freezing, Stress tolerance

## Abstract

**Background:**

Switchgrass (*Panicum virgatum*) is a warm-season perennial grass. Improving its cold tolerance is important for its sustainable production in cooler regions. Through genome-wide bioinformatic analysis of switchgrass *Zinc finger*-*CCCH* genes (*PvC3Hs*), we found that several *PvC3Hs*, including *PvC3H72*, might play regulatory roles in plant cold tolerance. The objectives of this study were to characterize *PvC3H72* using reverse genetics approach and to understand its functional role in cold signal transduction and cold tolerance in switchgrass.

**Results:**

*PvC3H72* is an intronless gene encoding a transcriptional activation factor. The expression of *PvC3H72* was rapidly and highly induced by cold stress. Transgenic switchgrass with over-expressed *PvC3H72* driven under maize ubiquitin promoter showed significantly improved chilling tolerance at 4 °C as demonstrated by less electrolyte leakage and higher relative water content than wild-type (WT) plants, as well as significantly higher survival rate after freezing treatment at − 5 °C. Improved cold tolerance of *PvC3H72* transgenic lines was associated with significantly up-regulated expression of *ICE1*–*CBF*–*COR* regulon and ABA-responsive genes during cold treatment.

**Conclusions:**

*PvC3H72* was the first characterized switchgrass cold-tolerance gene and also the only *Znf*-*CCCH* family gene known as a transcription factor in plant cold tolerance. PvC3H72 was an added signaling component in plant cold tolerance associated with regulation of ICE1–CBF–COR regulon and ABA-responsive genes. Knowledge gained in this study not only added another acting component into plant cold-tolerance mechanism, but also be of high value for genetic improvement of cold tolerance in switchgrass as well as other warm-season grasses.

## Introduction

Cold stress involves chilling stress and freezing stress, which is a primary factor limiting the growth of warm-season plant species [1, 2]. Chilling stress with temperature ranging from 0 to 15 °C causes cell membrane destabilization and metabolic dysfunction, including inhibition of photosynthesis, severe cellular dehydration and oxidative burst and freezing, which occurs at temperature below the freezing point of water, causes both intracellular and extracellular freezing and can cause lethal cellular damages [3, 4]. To survive through cold temperatures, plants evolved multifaceted signaling pathways to sense and transduce cold stress signals to re-program transcriptional pathways and activate downstream functional genes. One canonical cold signaling cascade is the *ICE1*–*CBF*–*COR* pathway involving ICE1 (Inducer of CBF expression) which is regulated by multiple post-translational modifications. Under cold stress, the sumoylated and phosphorylated ICE1 is stabilized, and positively regulates expression of *CBF*s (*C*-*repeat*-*binding factors*) by directly targeting the *ICE1* box of *CBF*s promoter and by inhibiting the transcription of *MYB15* which encodes a transcriptional suppressor upstream of *CBF*s [5–7]. CBFs directly bind to the conserved *CRT/DRE* cis-elements of their downstream cold-regulated genes’ (*COR*s) promoters [6]. *COR* genes encode functional proteins, such as dehydrins or late embryogenesis abundant (LEA) proteins for membrane stabilization, protecting protein stability and functionality from aggregation and against freeze–thaw inactivation [8–11].

CCCH-type zinc finger (Znf) proteins, with the character of three cysteines and one histidine residues, are a kind of proteins playing significant roles in plant growth, development, abiotic and biotic stresses [11–23]. A number of *CCCH* family genes, such as *AtTZF1*, *GhZFP1*, *OsTZF1*, *AtSZF1/2*, *OsDOS,* were found as important regulators for plant responses to salt, drought, and oxidative stresses [11–17]. In our previous study, genetic structure, functional motif, and gene expression pattern in 21 different organs/tissues were analyzed for switchgrass *CCCH* family genes [18]. According to the analysis, we hypothesized that eight switchgrass *PvC3H* genes classified in Clade-XIV were likely function as regulators in plant abiotic stress tolerance [18]. Several other genome-wide studies on *CCCH* family genes in rice (*Oryza sativa*), maize (*Zea mays*), Arabidopsis (*Arabidopsis thaliana*), *Medicago truncatula*, tomato (*Solanum lycopersicum*), and Chickpea (*Cicer arietinum*) also identified some novel stress-responsive *CCCH* genes [19–23]. However, there has been no report on *CCCH* type genes’ involvement in plant cold tolerance and signal transduction to date.

Switchgrass is a perennial C_4_ grass as a prime candidate for bioenergy feedstock production, which exhibits superior productivity in the long warm summers but sensitive to cold stress [1]. Improved cold tolerance is an important factor to consider when growing switchgrass as well as other warm-season plants to achieve superior biomass productivity in cool temperate regions [24]. In our genome-wide analysis of switchgrass *CCCH* genes, we found that a few of clade-XIV genes were cold responsive [18]. The objective of this study was to characterize cold-responsive gene *PvC3H72* using reverse genetics approach and to understand its functional role in cold signal transduction and cold tolerance in switchgrass.

## Results

### PvC3H72 identified as a novel zinc finger CCCH-type transcription factor

**Fig. 1:**
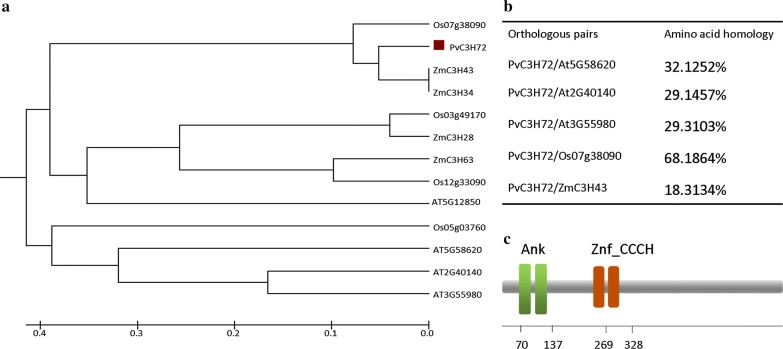
Phylogenetic tree, amino acid homology and protein structure analysis of PvC3H72 and its orthologous proteins. **a** Phylogenetic tree of PvC3H72 and its orthologous proteins in rice, maize and Arabidopsis. The evolutionary history was inferred using the UPGMA method with the sum of branch length = 3.14203139 using MEGA 5. The evolutionary distances were computed using the Poisson correction method and are in the units of the number of amino acid substitutions per site. **b** Amino acid homology between PvC3H72 and its orthologous proteins. **c** Structural annotation of PvC3H72 for the position of the two ANK domains (represented in green boxes) and two Zinc finger-CCCH motifs (brown boxes)

*PvC3H72* is intronless, encoding a deduced protein of 667 aa, with a pI of 6.55, molecular weight of 70.975 kD, two conserved Ankyrin repeats, and two zinc finger (Znf)-CCCH domains (Fig. 1). An UPGMA phylogenetic tree of PvC3H72 and their closest orthologous proteins in rice, maize and Arabidopsis were conducted (Fig. 1). Its closest orthologous proteins are ZmC3H43/34 in maize (noting that ZmC3H34 is an N’-truncated protein of ZmC3H43) and Os07g38090 in rice. Their closest Arabidopsis ortholog is an unknown protein At5g12850 with 34.3% protein sequence homology to PvC3H72. None of its orthologous genes has been functionally characterized before, indicating that *PvC3H72* is a novel zinc finger-*CCCH* family gene.

**Fig. 2:**
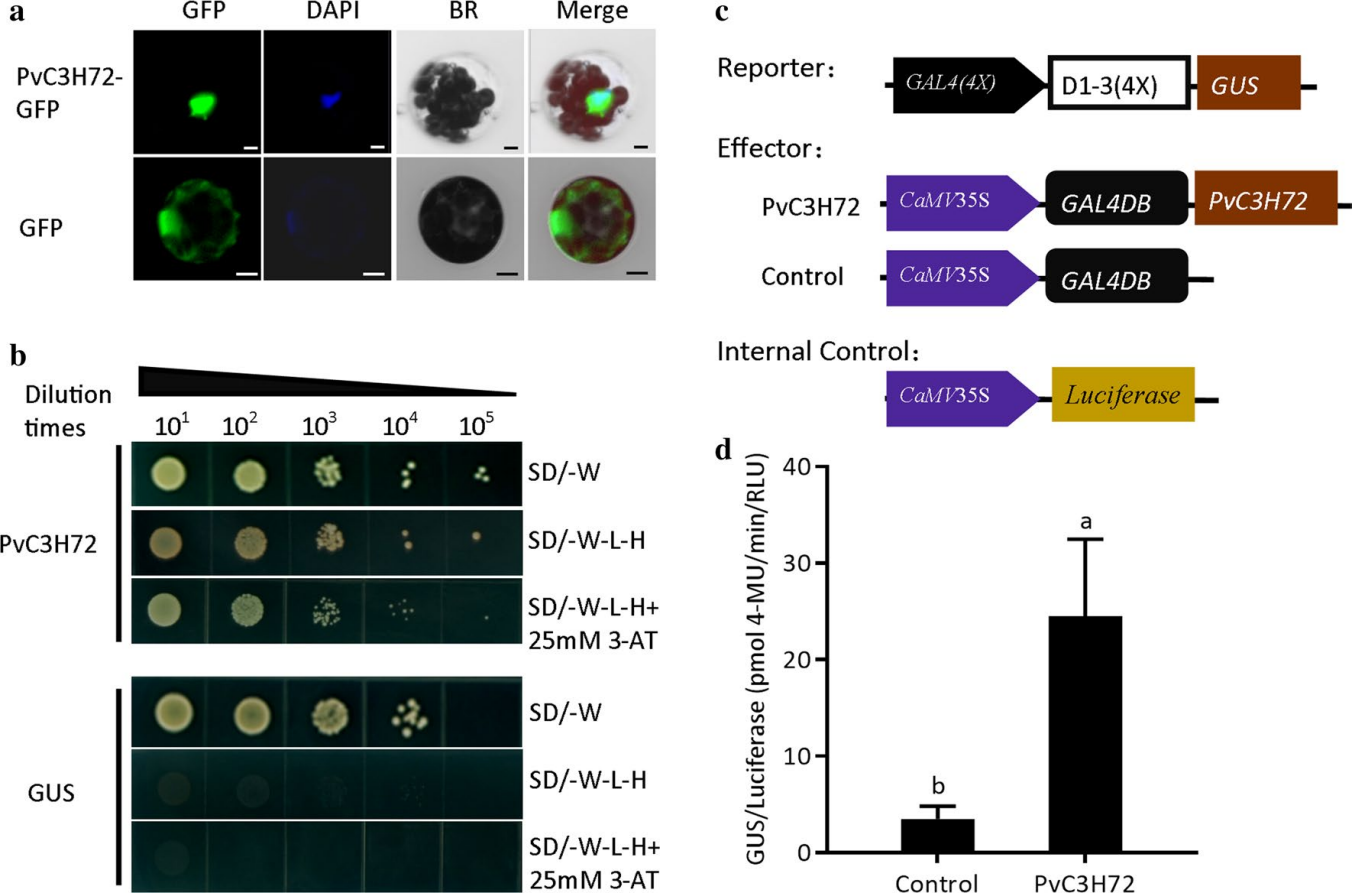
PvC3H72 subcellular localization and transcriptional activity assay. **a** Subcellular localization of PvC3H72–GFP fusion protein in Arabidopsis protoplast. GFP protein alone shows fluorescent signals in nucleus, membrane, and cytoplasm. DAPI is a staining dye for the nucleus. Bars = 5 µm. **b** PvC3H72 transcriptional activity in yeast system. GUS was used as a negative control. **c** Schematic diagram of three plasmids used for transactivation activity. Three plasmids (effector, reporter and internal control) were electroporated into Arabidopsis protoplasts at the ratio of 5:4:1. **d** PvC3H72 showed transactivation activity in the plant cells. All the experiments were repeated three times with similar results. Letters above bars indicate significant difference at *P* < 0.05 (*n* = 3)

Znf-CCCH family proteins were known to bind DNA and RNA through their Znf-CCCH domain(s). To observe the subcellular localization of PvC3H72, we fused the gene with GFP tag at the C-terminal and transformed the fusion gene into the Arabidopsis protoplast. It clearly showed that the PvC3H72-GFP fusion protein was solely localized within the DAPI-stained nucleus (Fig. 2a).

To further verify whether PvC3H72 is transcription factor, we performed yeast and plant cell transcriptional activity assays. As shown in Fig. 2b, yeast cells transformed with pGBKT7-*PvC3H72* vector, in which vector the gene was fused with the GAL4 DNA-binding domain (GAL4BD), could grow on the stringent selection medium SD/–W-L–H supplemented with 25 mM 3-AT by trans-activating the reporter genes in the yeast, while those transformed with the control vector pGBKT7-*GUS* (*UidA* gene as negative control) could not grow. Transcriptional activation of PvC3H72 was also verified in plant cells (Fig. 2c, d). Transient over-expression of PvC3H72-GAL4BD fusion protein activated the *GUS* reporter gene that was under driven of an artificial promoter containing four copies of GAL4 DNA-binding sites [GAL4(4x)-D1-3(4x)] with a significantly higher GUS/LUC ratio than that of the empty vector control. These results proved that PvC3H72 is a transcriptional factor with transactivation activity.

### *PvC3H72* transcriptionally responsive to cold stress

**Fig. 3:**
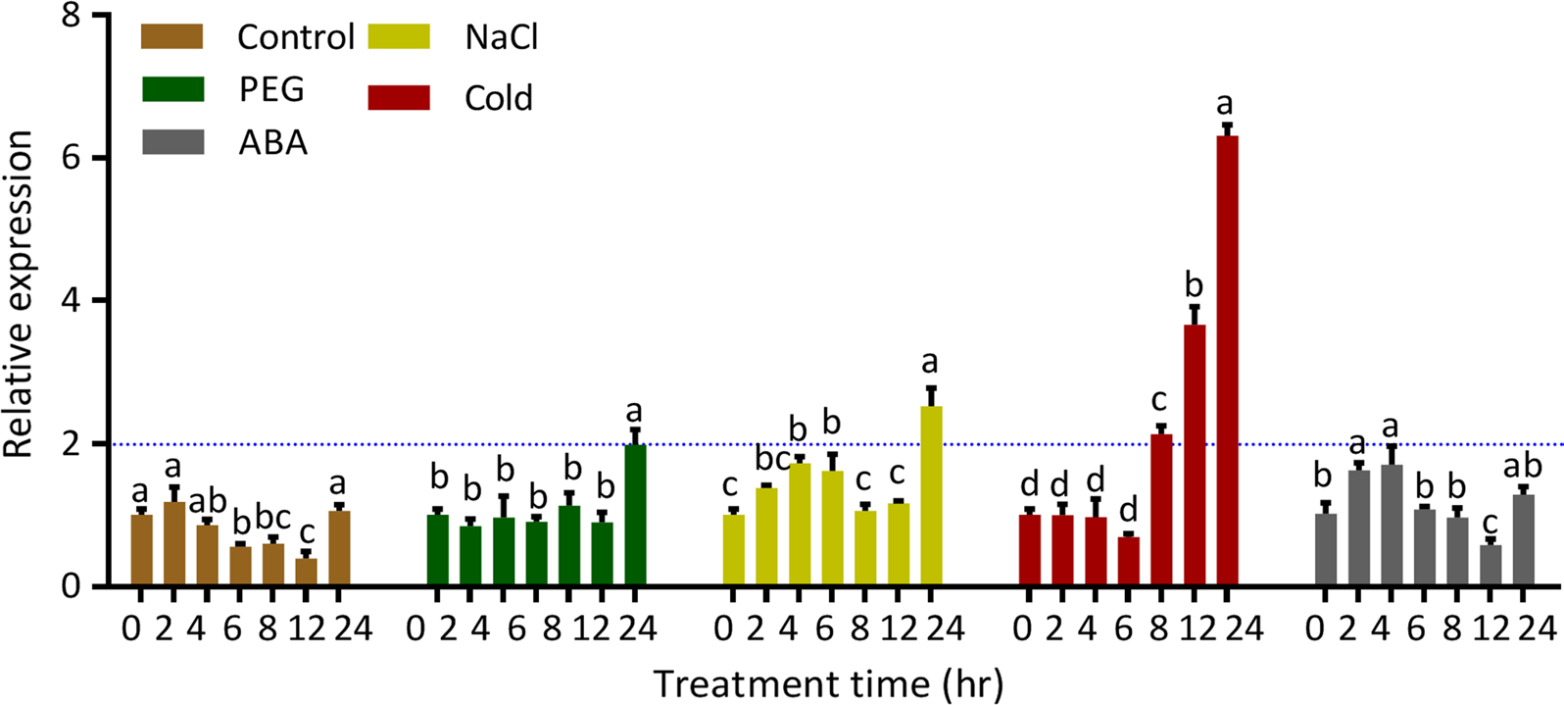
qRT-PCR analysis on relative expression of *PvC3H72* in response to different abiotic treatments. Four-week-old seedlings were treated with 100 µM ABA, 20% PEG6000 (w/v), NaCl (250 mM) or cold (4 °C). Letters above bars indicate significant difference at *P* < 0.05 (*n* = 3)

To examine whether *PvC3H72* was responsive to general abiotic stress, including cold, drought and salt stress, the relative expression pattern of *PvC3H72* under each stress was examined. *PvC3H72* was highly inducible by cold treatment, as its expression level increased by ~ 2-, 3.7- and 6.2-fold after 8, 12 and 24 h of cold treatment, respectively (Fig. 3). PvC3H72 was also responsive to PEG-induced drought and salt stress, with twofold up-regulated expression after 24 h of treatment. The expression of *PvC3H72* was responsive to abiotic stress, particularly more responsive to cold stress with higher expression levels than with PEG and NaCl after 24 h of treatment.

### Overexpression of *PvC3H72* improved switchgrass tolerance to chilling and freezing stress

**Fig. 4:**
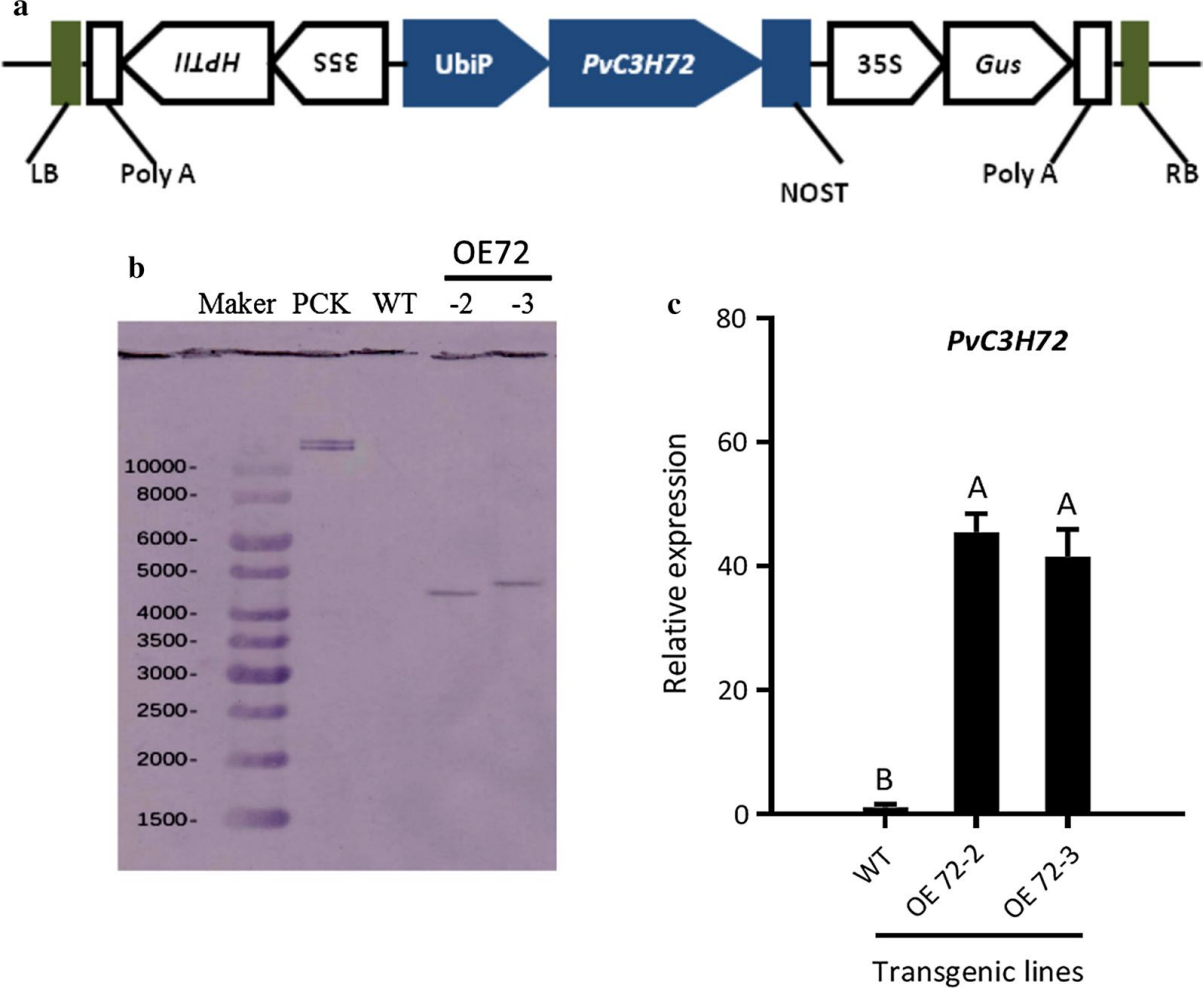
Confirmation of *PvC3H72*-overexpression (OE-72) transgenic lines. **a** Schematic diagram of T-DNA used for genetic transformation. **b** Southern blot for WT and two OE-72 transgenic lines. The binary vector used for transformation was the positive control (PCK). **c** Relative expression of *PvC3H72* in WT and two OE-72 lines using qRT-PCR. Letters above bars indicate significant difference at *P* < 0.05 (*n* = 3)

To further characterize the function of *PvC3H72*, we generated transgenic switchgrass plants with the gene driven under maize ubiquitin promoter. *UidA* (conferring GUS) and *HPTII* (conferring hygromycin resistance in the selection medium) genes were used as selection marker genes flanking the target gene (Fig. 4a). *PvC3H72*-overexpression (OE72) transgenic lines were verified by GUS staining and PCR (Additional file 1: Figure S1). Southern blot showed that the two transgenic lines (OE72-2 and OE72-3) were resulted from distinct transgenic events with single T-DNA insertion (Fig. 4b). Under normal growth condition, these OE transgenic lines did not show significant phenotypic alteration compared to the wild-type (WT) plants (Additional file 2: Table S1). We selected two transgenic lines (named as OE72-2 and -3) for further analysis. The relative expression of *PvC3H72* of these two lines was checked using qRT-PCR that showed ~ 42 and 38 times higher expression levels than that in the WT plants (Fig. 4d).

**Fig. 5:**
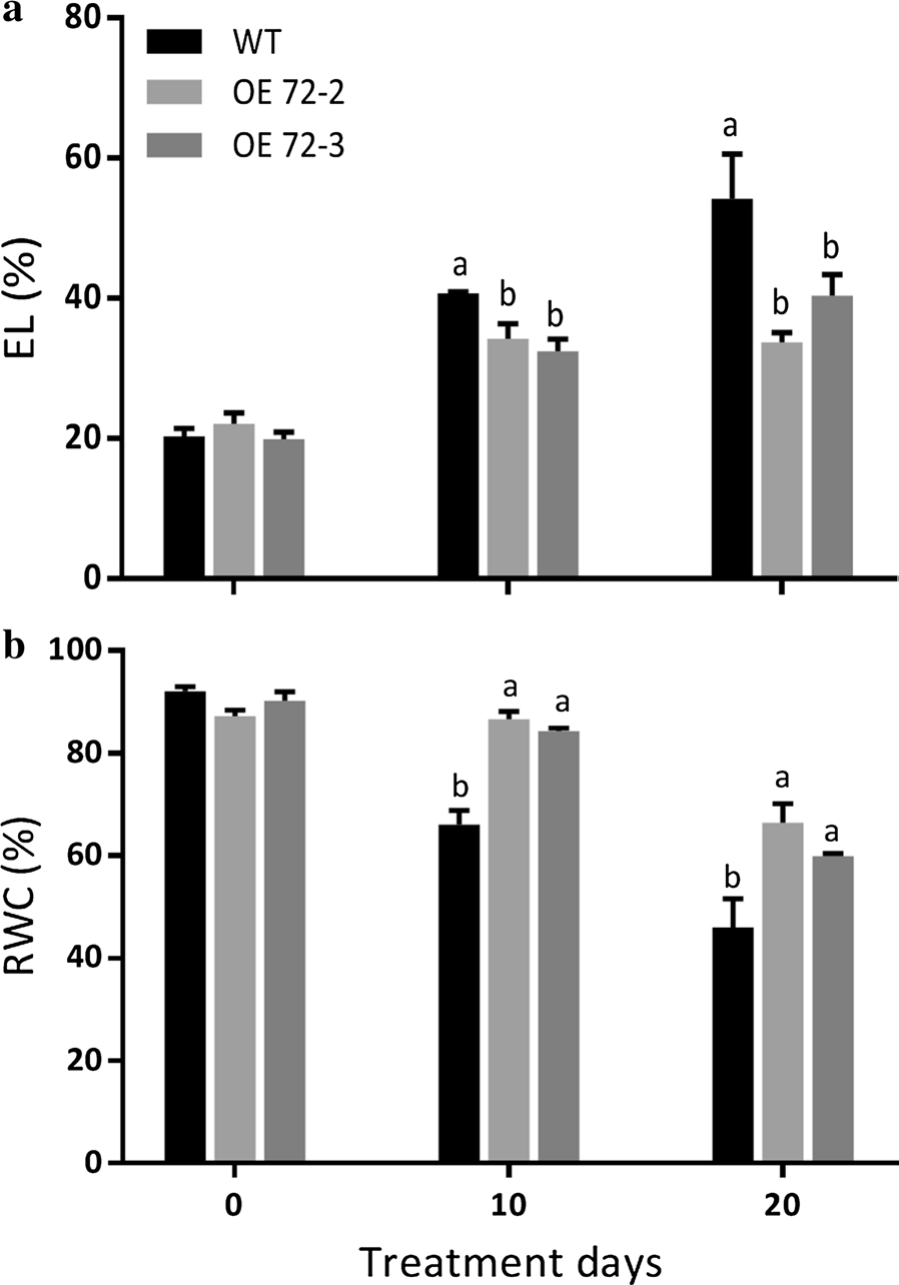
*PvC3H72*-overexpression (OE-72) transgenic lines demonstrated lower Electrolyte leakage (EL) and higher Relative water content (RWC) after chilling treatments at 4 °C. Mean and SD values were obtained from more than three replicates. All experiments were repeated three times. Letters above bars indicate significant difference at *P* < 0.05 (*n* = 4 replicates)

To characterize whether *PvC3H72* play regulatory roles in switchgrass chilling tolerance, we compared the fitness of non-acclimatized WT switchgrass with OE72 transgenic lines under 4 °C for 20 days. As shown in Fig. 5, WT and all tested transgenic lines had similar RWC and EL values before the chilling treatment. After 10 and 20 days of chilling treatment, OE72 lines showed higher RWC but lower EL than WT plants. And the difference of the EL value between WT and OE72 transgenic lines became even greater after 20 days of chilling treatment.

**Fig. 6:**
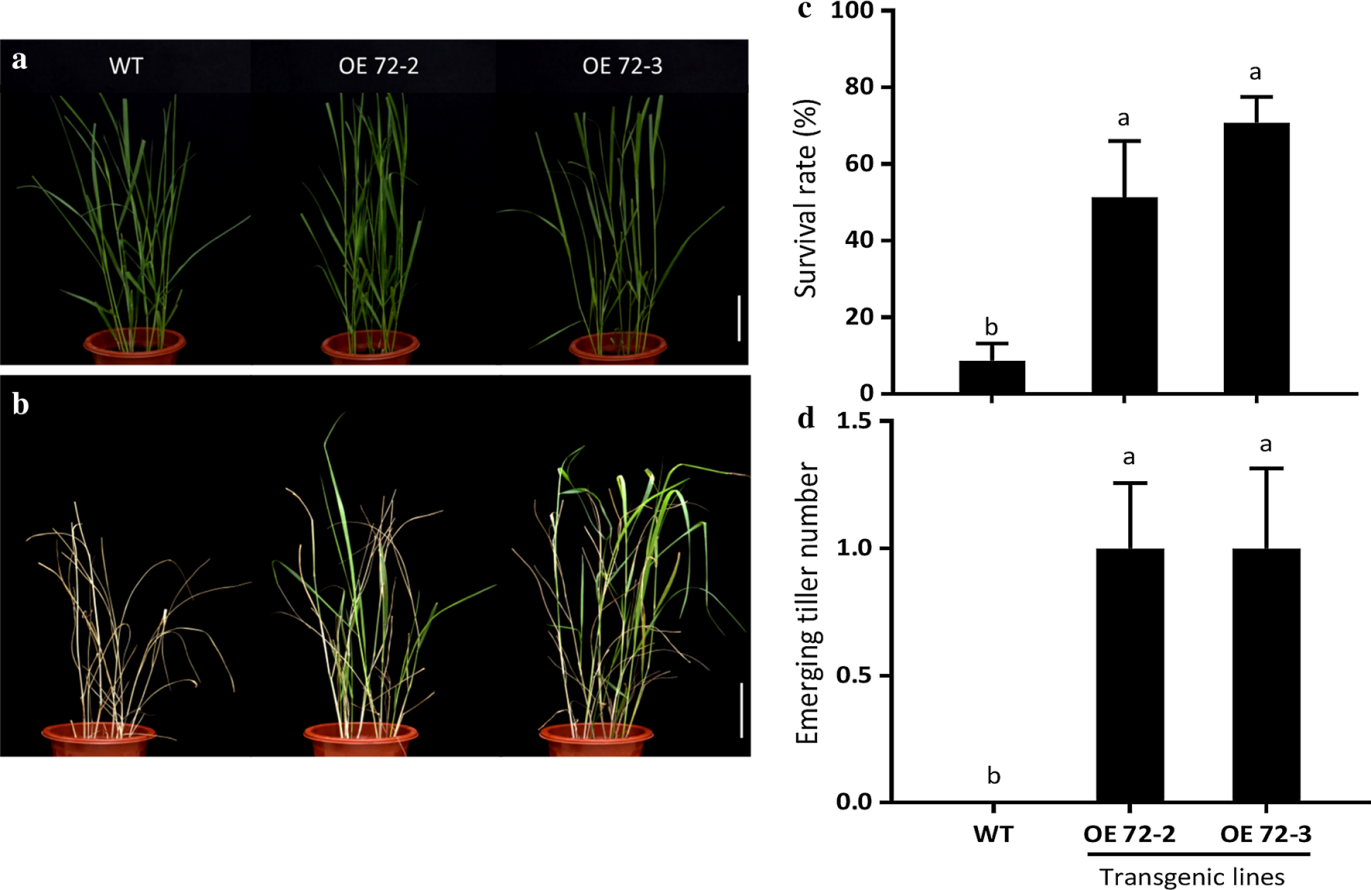
*PvC3H72*-overexpression (OE-72) transgenic lines were more tolerant to freezing stress (at − 5 °C). Phenotype of OE-72 and WT plants under control condition (**a**) or after freezing treatment (**b**). **c** Survival rates after freezing treatments. **d** Number of newly emerged tillers after recovery. All experiments were repeated three times. Letters above bars indicate significant difference at *P* < 0.05 (*n* = 9 replicates)

Cold-acclimatized OE72 and WT plants were treated at − 5 °C for freezing tolerance test. As shown in Fig. 6, freeze-treated WT plants exhibited severer visible damage and had significantly lower survival rate and newly emerged tiller numbers than two OE72 transgenic lines. The result showed that over-expressing *PvC3H72* significantly improved switchgrass freezing tolerance, as shown by increased survival rate up to 50–68% (Fig. 6).

### Overexpression of *PvC3H72* led to increased expression of *ICE1*–*CBF*–*COR* transcriptional cascade genes

**Fig. 7:**
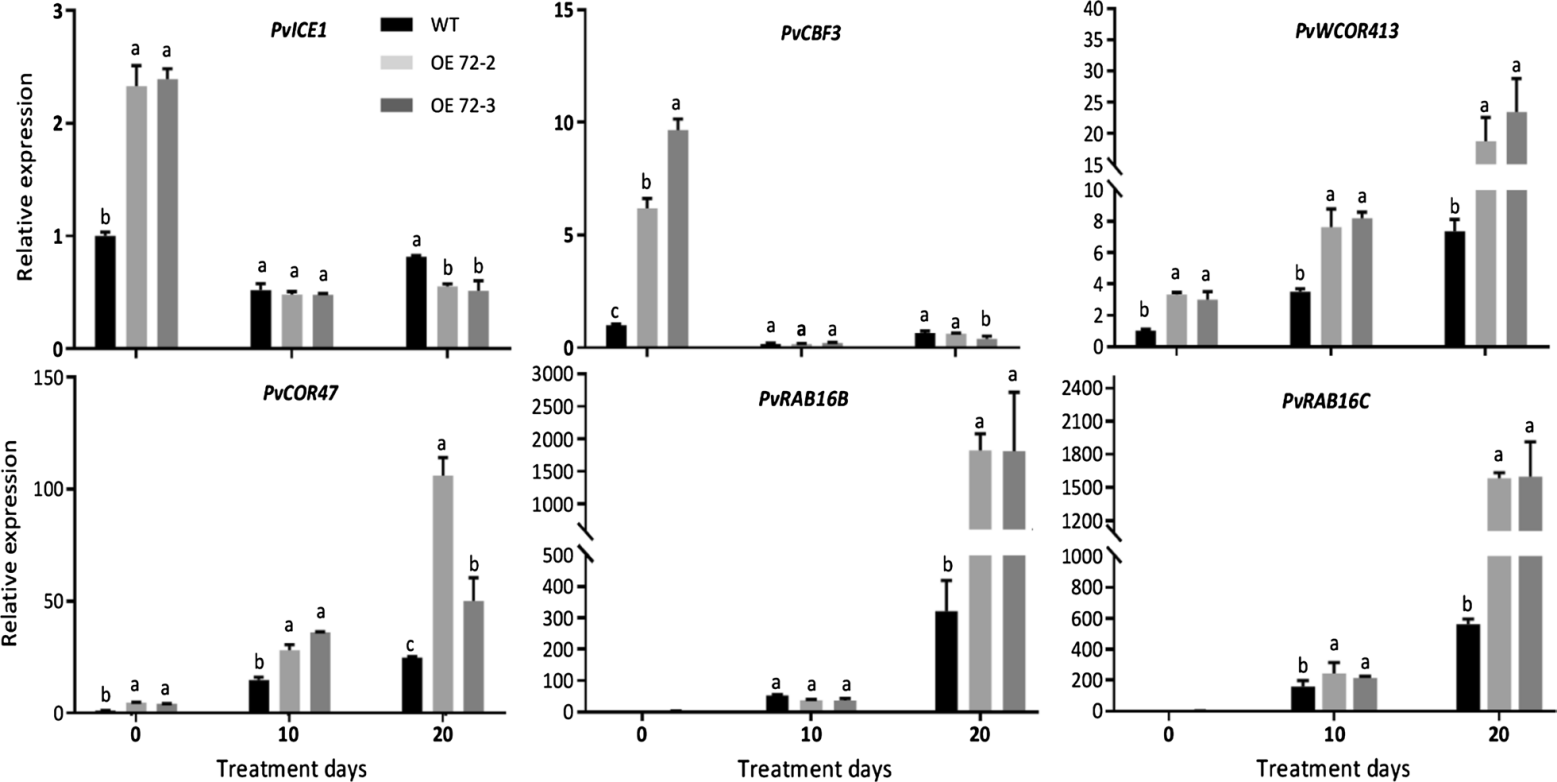
Relative expression of selected cold-tolerance genes during chilling treatment. Relative expression of putative switchgrass *ICE1*–*CBF*–*COR* regulon genes (*PvICE1*, *PvCBF3*, *PvWCOR413* and *PvCOR47*) and two ABA-responsive polypeptide-encoding genes (namely, *PvRAB16B* and *PvRAB16C*) were analyzed using qRT-PCR. Letters above bars indicate significant difference at *P* < 0.05 (*n* = 3 replicates)

To examine whether improved cold (chilling and freezing) tolerance in OE72 transgenic plants was associated with alteration in ICE1–CBF–COR transcriptional cascade genes, we further characterized relative expression levels of *PvICE1*, *PvCBF3*, two cold-responsive (*COR*) genes (namely, *PvCOR47*, *PvWCOR413*) and two ABA-responsive polypeptide-encoding genes (namely, *PvRAB16B* and *PvRAB16C*) in WT and transgenic switchgrass lines before and after 10 or 20 days of chilling (4 °C) treatment. As shown in Fig. 7, expression levels of *PvICE1* and *PvCBF3* were significantly higher in two OE72 transgenic lines than in WT before chilling treatment. Consistently, *PvCOR47* and *PvWCOR413*, which were putative direct downstream genes of CBF transcription factors, also had significantly higher expression levels in two transgenic lines than in WT before and after the treatment. On the other hand, expression levels of two ABA-responsive genes (*PvRAB16B* and *PvRAB16C*) were only significantly higher in transgenic lines after 20 days of chilling treatment. This result showed that over-expressing *PvC3H72* not only significantly increased the expression of *ICE1*–*CBF*–*COR* transcriptional cascade genes but also increased ABA-responsive polypeptide-encoding genes at the late stage of chilling treatment.

## Discussion

Plant Znf-CCCH proteins are implicated in various biological processes, including development and cell fate specification [25–28], plant biotic defense [29], and stress tolerance [16, 17, 28, 30, 31], and their functions were often through RNA-binding, protein–protein binding and DNA-binding [11, 32]. PvC3H72 has two conserved Ankyrin repeats besides the Znf-CCCH domain but does not have any known RNA-binding domain (e.g., RNA-Recognition Motif or K homolog domains) (Fig. 1). PvC3H72 was localized in nucleus and also had transcriptional activity (Fig. 2), confirming that PvC3H72 was a transcription factor. The Ankyrin repeat domain is one of the most common protein–protein interaction motifs in nature having been identified in proteins of diverse functions, including transporters, cytoskeletal and transcriptional initiators, etc. [33]. Further identification of interacting proteins of PvC3H72 may provide further information on the signaling cascade in which PvC3H72 is involved.

To date, no *CCCH* gene has been identified as plant cold-tolerance regulators before. Our genome-wide identification and analysis of switchgrass *CCCH* family genes predicated that clade-XIV *PvC3H* genes might be involved in plant stress tolerance [18]. This functional characterization on *PvC3H72* provided experimental evidence supporting our previous hypothesis. To date, none of the *PvC3H72*’s closet orthologs in other plant species (e.g., Arabidopsis, rice, and maize) has been functionally characterized yet. Another two Arabidopsis orthologous proteins of PvC3H72 are At2G40140 (AtSZF2) and At3G55980 (AtSZF1) with only 29.1% and 29.3% amino acid identity, respectively (Fig. 1a). *AtSZF1* and -*2* are quickly and highly responsive to salt stress and their over-expression lines were more tolerant to salt tolerance. However, *PvC3H72* was not quickly or highly induced by salt treatment (less than twofold within the first 12 h of NaCl treatment), and our preliminary experiments on salt- and dehydration-treated transgenic lines did not show any noticeable difference at all (data not shown). Over-expressing *PvC3H72* significantly improved switchgrass freezing and chilling tolerance that cold acclimatized transgenic plants had ~ 50–68% survival rate compared to less than 10% of the WT plants after freezing treatment, and transgenic switchgrass also showed better leaf water status and cell membrane integrity during chilling treatment. To our best knowledge, *PvC3H72* was the first identified plant *Znf*-*CCCH* gene positively regulating switchgrass cold tolerance.

Perception and signal transduction pathways have been well studied mainly in model plants Arabidopsis and rice. Upon the onset of cold stress, membrane proteins such as COLD1/RGA1 and other components (e.g., Ca^2+^ channels) are activated, leading to an influx of Ca^2+^, production of reactive oxygen species, accumulation of ABA and activation of mitogen-activated kinase (MAPK) cascade. The main signal cascade in nucleus is the ICE1–CBFs–COR signaling pathway. In this pathway, cold-induced ICE1 acts in the upstream and precedes the induced expression of *CBF*s, and the induced expression of *CBF*s causes the increased expression of *COR* genes (and therefore, expression of *CBF*s is an earlier event than induction of *COR*s) [34]. Upon cold stress perception, ICE1 was phosphorylated and stabilized through the MAPK cascade, and ICE1 rapidly induces the expression of *CBF/DREB1s* to trans-activate the expression of downstream *COR* genes by direct recognizing and binding to the conserved *CRT* cis-elements in their promoters [6, 35, 36]. *COR* genes encode extremely hydrophilic proteins, most of which are members of the dehydrins or LEA (late embryogenesis abundant) proteins or with unknown functions [37]. LEA proteins are important for membrane stabilization, protecting protein stability and functionality from aggregation and against freeze–thaw inactivation [8, 10, 11]. For example, OsWCOR413 is a thylakoid transmembrane protein supporting membrane stability and possibly a receptor of cellular signal in cold acclimation [38]. Over-expressing *OsWCOR413* provided higher cold tolerance in rice [39]. *COR47* encodes a dehydrin protein, the expression of which was cold inducible. The Arabidopsis dehydrin COR47 showed cryoprotective activity of thylakoid membranes during freeze–thaw cycles [40]. Expression of *PvCOR47* and *PvWCOR413* is also inducible at the mRNA level [41]. In this study, we found that the OE72 lines had significantly increased expression of *ICE1*–*CBF*–*COR* transcriptional cascade genes (*PvICE1*, *PvCBF3*, *PvWCOR413* and *PvCOR47*). It was notable that *ICE1* expression at mRNA level was not inducible by cold stress [42], yet over-expressing *ICE1* in Arabidopsis, cucumber (*Cucumis sativus*) and rice all improved plant cold tolerance [43, 44]. And switchgrass cold-responsive *CBF/DREB1* family genes were rapidly but only transiently increased upon cold stress [45]. Our result was consistent with these previous findings that expression levels of *PvICE1* and *PvCBF3* was higher in OE72 lines which might be attributed to better chilling tolerance in these lines; while expression of *PvICE1* and *PvCBF3* in OE72 lines reduced to similar levels of those in WT after prolonged exposure to cold that could be beneficial to the maintenance of a balanced energy consumption between stress tolerance and growth and development needs.

Although cold effects on the induction of gene expression were mainly through the ABA-independent pathway [2], alternative cold signaling pathways in the nucleus also include activated transcription factors through a series of Ca^2+^-binding proteins, and ABA signaling pathway transduced by transcription factors (e.g., ABF1/2) and their down-stream ABA-responsive genes [2]. ABA-responsive genes, *RAB16B* and *RAB16C*, are late drought- and cold stress-responsive genes [46], and we also found that these downstream ABA-responsive polypeptide-encoding genes were only up-regulated in transgenic lines at late stage of chilling treatment. Nevertheless, the exact underlying molecular mechanism regulating *PvC3H72*-involved cold acclimation is to be identified in the future.

## Conclusion

Over-expressing *PvC3H72* improved chilling and freezing tolerance in switchgrass. PvC3H72 could be used as a target gene in genetic modification or as a molecular marker for improving warm-season plant tolerance to cold stress. Further analysis of up-stream factors or signaling molecules regulating the responsiveness of PvC3H72 to cold stress could provide insightful information on molecular mechanisms of PvC3H72 regulation of cold stress.

## Experimental procedures

### Gene cloning and vector construction

*PvC3H72* (Phytozome accession no.: Pavir.J07041.1) was previously identified as a *CCCH*-type Znf family gene and classified in the clade-XIV as a stress-responsive gene [18]. The full length gene was amplified from the gDNA of a selected line ‘HR8’ from switchgrass lowland ecotype ‘Alamo’ [47], and its encoded amino acid sequence is shown in Additional file 3: Figure S2. The gene was cloned into the Gateway entry vector pENTR/D (Invtirogen, Carlsbad, CA). The gene was subcloned into p2GWF7 [48], pGBKT7 (Invitrogen) and pVT1629 [47] through LR reaction (Invitrogen).

### Plantlets treatment for expression analysis of *PvC3H72*

Four-week-old switchgrass plantlets grown in 1/2 Hoagland nutrient solution were put under cold treatment (4  °C) or treated by adding 20% (w/v) polyethylene glycol (PEG)-6000, 250 mM NaCl, or 100uM ABA in the hydroponic culture. Then, the treated plantlets were sampled after 0, 1, 2, 4, 8, 12 and 24 h of treatment for PvC3H72 expression analysis using qRT-PCR.

### Observation of subcellular localization of PvC3H72-GFP

The *PvC3H72* was subcloned into a modified gateway-compatible P2GWF7.0 vector to put *PvC3H72* in fusion with *GFP*. By polyethylene glycol (PEG)-mediated Arabidopsis protoplast transformation [49], the *PvC3H72*-*GFP* fusion gene was overexpressed in Arabidopsis protoplasts. DAPI were used to stain the nucleus, and the GFP signal were detected under a Zeiss LSM 780 laser scanning confocal microscope (Carl Zeiss SAS, Jena, Germany).

### Transactivation assay

The *PvC3H72* was subcloned into the BD vector pGBKT7 to fuse PvC3H72 with the DNA-binding domain of GAL4. The pGBKT7-*PvC3H72* and the control vector pGBKT7-*GUS* (*UiDA gene*) were then transformed into the yeast strain Y2HGold (Clonetech, Mountain View, CA), separately. The transformed positive clones grown well on SD/-Trp were then grown on plates containing SD/-Trp-Leu-His and SD/-Trp-Leu-His + 25 mM 3-AT for auto-transactivation assay.

For the transcriptional activity assay of PvC3H72 in plant cells, *PvC3H72* was cloned into the 35S promoter-driven pZB370 vector to fuse with the yeast GAL4 DNA-binding domain (GAL4BD) as effector (pZB369-*PvC3H72*); while the vector without the target gene was used as the negative control. The internal control vector was pZB371-Luciferase with the *luciferase* reporter gene under driven of the 35S promoter as well. The reporter vector (pZB370-GUS) was constituted of four copies of GAL4 DNA-binding sites (GAL4(4x)-D1-3(4x)) to drive the *GUS* (*UidA*) reporter gene. Three plasmids (effector, reporter and internal control) were electroporated into Arabidopsis protoplasts at the ratio of 5:4:1. The transcriptional ability of PvC3H72 was assessed by the GUS/LUC ratio. Three biological replicates were included for each combination.

### Switchgrass genetic transformation and transgenic plant verification

According to the protocol described previously [47, 50], we infected embryogenic calluses of a switchgrass line ‘HR8’ with *Agrobacterium tumefaciens* strain ‘AGL1’ harboring the binary vector pVT1629-*PvC3H72* with the target gene under driven of the maize *ubiquitin* promoter, and selected the putative transgenic lines on 50 mg/L hygromycin (Sigma-Aldrich, St. Louis, MO, USA). The rooted hygromycin-resistant plantlets were transplanted into soil and further checked by GUS staining and regular PCR for the presence of the T-DNA fragment. The Southern blot experiment was performed as reported before [50]. Switchgrass is a self-incompatible allotetraploid. Seeds of two selected lines were from a crossing between the transgenic lines with WT and their segregation ratios were further checked by GUS staining. T1 seeds with two transgenic lines with a segregation ratio of about 1:1 were used for the experimental analysis in the study.

### Chilling and freezing treatments

Two-month-old plants of switchgrass at the vegetative stage cultivated in a greenhouse were transferred to a growth chamber (SaiFu limited company, Ningbo, China, ZRX-1100G) with temperature controlled at 25/23 °C (day/night), 50% humidity, and 680 µmol/m^2^/s photosynthetically active radiation at the canopy level with 12 h of light period a day. Plants were maintained in the growth chamber for 2 weeks for acclimation to the growth chamber conditions prior to be exposed to cold stress. For chilling stress, plants were grown at 4 °C (day/night) for 20 days. For freezing treatment, plants were first acclimated to 4 °C (day/night) for 20 days. Acclimated plants were placed in a freezer with temperature initially set at − 1 °C and then gradually decreased to − 5 °C within 6-hour period. Plants were maintained in − 5 °C for 27 h for freezing stress, and then gradually increased to 4 °C in 6 h. After 8 h in 4 °C, the freezing-treated plants were moved to the growth chamber with temperature controlled at 25/23 °C for 3 weeks for the evaluation of survival from freezing stress. Newly emerged tillers from each plant were counted. Each stress treatment was repeated in replicates of nine plants.

### Electrolyte leakage and relative water content measurements

Two commonly used physiological indicators for stress tolerance, electrolyte leakage (EL) and relative water content (RWC), were measured to evaluate cold tolerance of both WT and transgenic plants. For EL measurement, 0.2 g of second-fully expanded leaves of a plant were rinsed three times, and then incubated in a 50-mL tube containing 35 mL of de-ionized water with constant shaking for 24 h. The initial conductance (*C*_i_) of the incubation solution with fresh leaves was measured using a conductivity meter (Thermo Scientific Orion, Model: Star A212, Cambridge, Mass., USA). The maximum conductance (*C*_max_) of the same incubation solution with leaves killed in boiling water was also measured. EL was calculated as *C*_i_/*C*_max_ expressed in percentage [51]. For RWC measurement, fresh leaves were cut into 1-cm-long leaf segments with their fresh weight (FW) weighed immediately, and then they were placed into tubes filled with deionized water for 12 h in dark at 4  °C to fully rehydrate the leaves to determine turgid weight (TW). Leaves were then dried in an oven at 80  °C for at least 72 h for dry weight (DW) measurement. The RWC was determined based on the formula: RWC (%)  =  [(FW  −  DW)/(TW  −  DW)]  ×  100 [52].

### Real-time quantitative reverse transcription PCR (qRT-PCR)

For qRT-PCR analysis, total RNA of switchgrass was extracted using RNApure fast isolation Kit (OMEGA, China) and the first strand cDNA was synthesized using the PrimeScript RT reagent Kit with gDNA Eraser (Takara, Otsu, Japan). PCR reactions were performed in triplicate with SYBR Green I Master reaction system (Roche Diagnostic, Rotkreuz, Switzerland) on Roche LightCycler480 II (Roche Diagnostic, Rotkreuz, Switzerland). Three biological repeats and two technical replicates were carried out. Data were determined by 2^−ΔΔCT^ calculation methods and normalized refer to the expression level of *PvFTSH4* as internal control according to Huang et al. [3]. Primers for qRT-PCR are listed in Additional file 4: Table S2.

### In silico and statistical analyses

Calculation of pI of the protein was conducted using the ExPASy server (http://web.expasy.org/computepi/). Motif prediction was conducted using the online SMART software (http://smart.embl-heidelberg.de/). The UPGMA phylogenetic tree was constructed using MEGA 5.0 software [53] after multiple sequence alignment using ClustalW (Additional file 5: Figure S3). Statistical analysis of the data was carried out by Fisher’s protected LSD at the probability of 0.05 using SAS v9.2 (SAS Institute, Cary, NC, USA).

## Supplementary information

**Additional file 1: Figure S1**. GUS staining and PCR verification of transgenic lines.

**Additional file 2: Table S1** Growth phenotypic data of WT and transgenic lines.

**Additional file 3: Figure S2** Sequence and functional motifs of PvC3H72.

**Additional file 4: Table S2** Primers used in this study.

**Additional file 5: Figure S3** Multiple sequence alignment of PvC3H72 and its orthologous proteins by ClustalW (B).

## Data Availability

All data generated or analysed in the present study are included in this published article and in additional information.
