# Supplementary figures and images for: Improved cold tolerance in switchgrass by a novel CCCH-type zinc finger transcription factor gene, PvC3H72, associated with ICE1–CBF–COR regulon and ABA-responsive genes
Source: Biotechnol Biofuels. 2019 Sep 20;12:224. doi: 10.1186/s13068-019-1564-y (PMC6753611; doi:10.1186/s13068-019-1564-y)

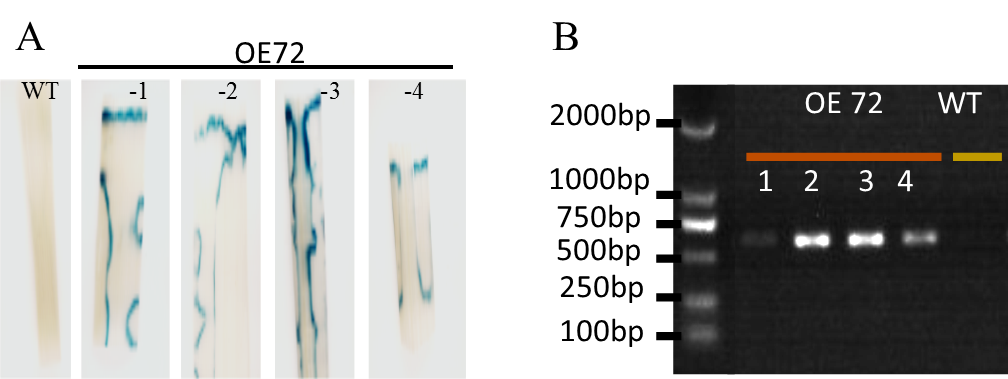

Supplement: Supplementary file 1 — Additional file 1: Figure S1. GUS staining and PCR verification of transgenic lines. [file 13068_2019_1564_MOESM1_ESM.tif]

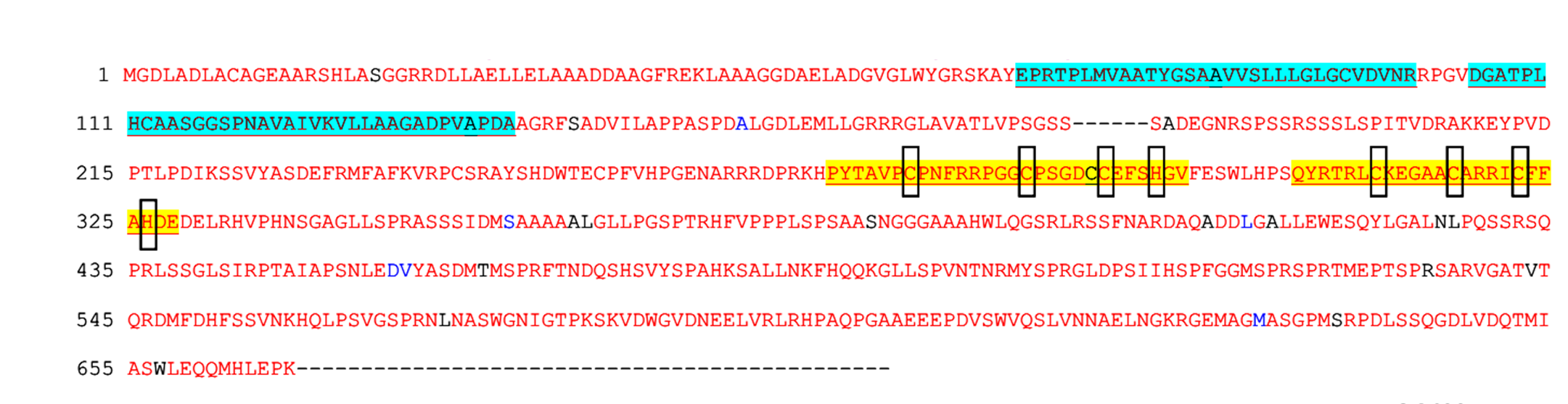

Supplement: Supplementary file 3 — Additional file 3: Figure S2 Sequence and functional motifs of PvC3H72. [file 13068_2019_1564_MOESM3_ESM.tif]

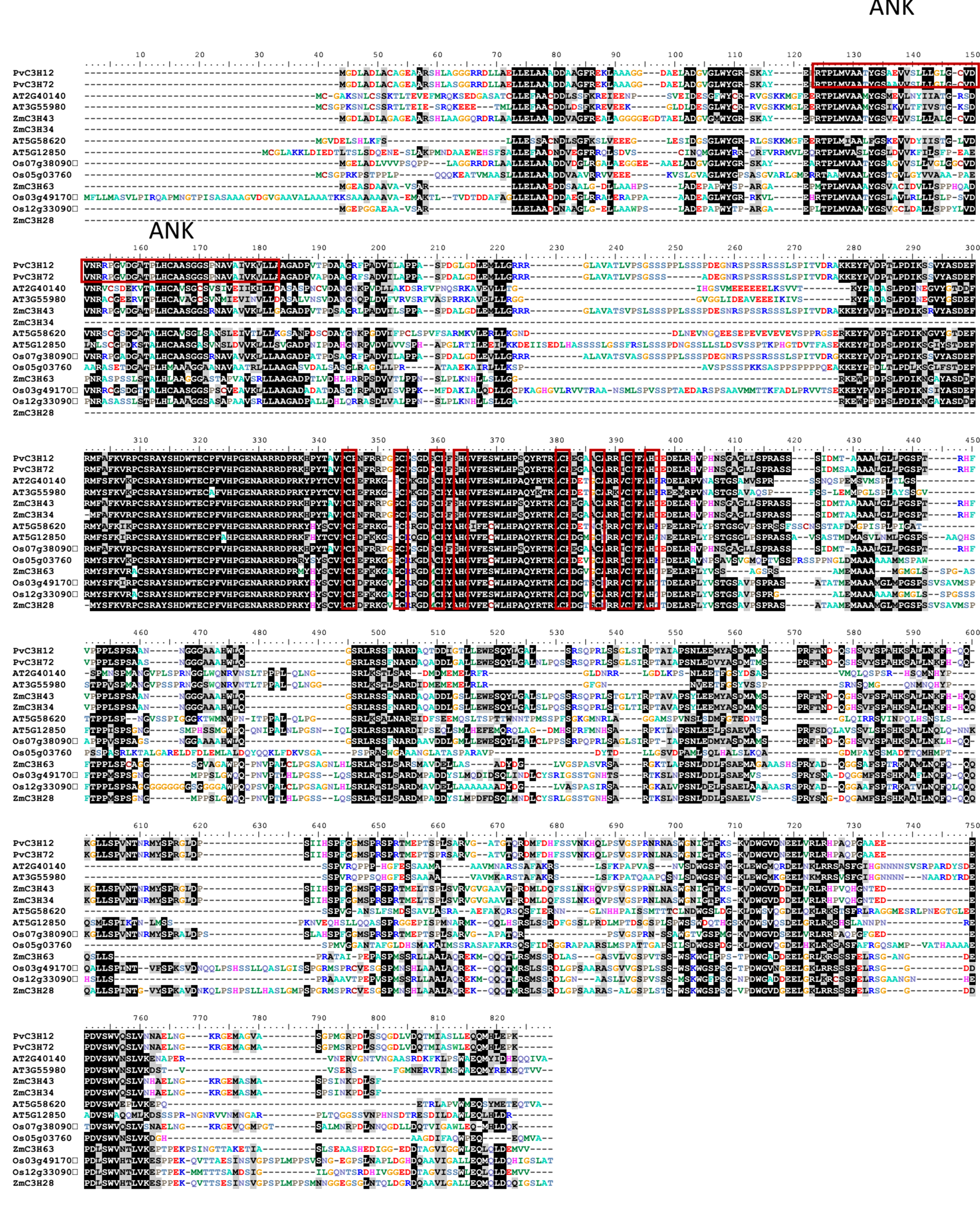

Supplement: Supplementary file 5 — Additional file 5: Figure S3 Multiple sequence alignment of PvC3H72 and its orthologous proteins by ClustalW (B). [file 13068_2019_1564_MOESM5_ESM.tif]
