# Supplementary material for: Improved cold tolerance in switchgrass by a novel CCCH-type zinc finger transcription factor gene, PvC3H72, associated with ICE1–CBF–COR regulon and ABA-responsive genes
Source: Biotechnol Biofuels. 2019 Sep 20;12:224. doi: 10.1186/s13068-019-1564-y (PMC6753611; doi:10.1186/s13068-019-1564-y)
Supplement: Supplementary file 2 — Additional file 2: Table S1 Growth phenotypic data of WT and transgenic lines. [file 13068_2019_1564_MOESM2_ESM.docx]

**Table S1 Growth phenotypic data of WT and transgenic lines**

| **Physiological index** | | **Wild type (WT)** | **OE72-2** | **OE72-3** |
| --- | --- | --- | --- | --- |
| Fresh weight (g) | | 104.82±6.387 a | 101.12±13.44 a | 91.68±4.43 a |
| Plant height (cm) | | 125.43±5.99 a | 114.00±3.14 a | 129.13±5.99 a |
| Tiller number | | 17.66±1.33 a | 18.160±1.60 a | 10.5±0.40 b |
| Node number | | 5.82±0.26 ab | 6.29±0.28 a | 5.17±0.34 b |
| Internode length (cm) | 1^st^ | 12.50±0.48 b | 11.41±0.36 b | 14.36±0.48 a |
|  | 2^nd^ | 14.34±0.58 b | 12.91±0.64 b | 16.39±0.75 a |
|  | 3^rd^ | 18.76±0.88 a | 14.86±0.69 b | 19.42±1.04 a |
|  | 4^th^ | 16.53±0.76 a | 14.61±0.88 a | 16.76±0.92 a |
| Stem diameter (mm) | 1^st^ | 4.05±0.13 a | 3.53±0.14 b | 3.56±0.13 b |
|  | 2^nd^ | 4.59±0.12 a | 3.70±0.11 b | 4.25±0.15 a |
|  | 3^rd^ | 5.09±0.12 a | 3.82±0.12 c | 4.48±0.16 b |
|  | 4^th^ | 4.65±0.14 a | 3.58±0.14 b | 4.51±0.19 a |
| Leaf length ( cm) | | 50.21±1.55 a | 49.91±0.96 a | 52.47±0.81 a |
| Leaf width (cm) | | 1.15±0.02 a | 1.11±0.02 a | 1.16±0.01 a |

Phenotypic data were obtained from switchgrass plants grown under normal growth grown in pots. Mean and SD values of fresh weight and plant height were from six replicates, while those of tiller number, node number, internode length, stem diameter, leaf length and leaf width were from more than sixteen replicates. The 1^st^( first), 2^nd^ (second),3^rd^ (third )and 4^t h^ (fourth) represent the order of internode or stem from the top to bottom, Letter indicates significant difference from water control at P < 0.05, using the Student’s t test.
