# Supplementary material for: Improved cold tolerance in switchgrass by a novel CCCH-type zinc finger transcription factor gene, PvC3H72, associated with ICE1–CBF–COR regulon and ABA-responsive genes
Source: Biotechnol Biofuels. 2019 Sep 20;12:224. doi: 10.1186/s13068-019-1564-y (PMC6753611; doi:10.1186/s13068-019-1564-y)
Supplement: Supplementary file 4 — Additional file 4: Table S2 Primers used in this study. [file 13068_2019_1564_MOESM4_ESM.docx]

| Target gene | Accession No. for the target gene | Primer sequence (5’-3’) | Purpose |
| --- | --- | --- | --- |
| *PvICE1* | Pavir.Ha01426.1 | TCCTTGGTGATGCAATTGAG | qRT-PCR |
|  |  | GGATGAAAGCTGGCTGATGT |  |
| *PvCOR47* | Pavir.J04551.1 | GTGGGAACGCACGCTTAC | qRT-PCR |
|  |  | CGACCTTCTGGACGACGA |  |
| *PvCBF3* | Pavir.Ga00931.1 | CCTGTGGAGCTACTGACGAT | qRT-PCR |
|  |  | CTCCTCTGCTCCTCCTGAT |  |
| *PvWCOR413* | Pv.J37131.1 | CGGGTTTCTCGCTTTTCAAC | qRT-PCR |
|  |  | GCACACCAAGGTACATCAGT |  |
| *PvRAB16B* | Pavir.Ab01356.1 | GCCCGGACAGCACTAATTAA | qRT-PCR |
|  |  | AACTACACTGCACACACACA |  |
| *PvRAB16C* | Pavir.J14171.1 | GGGCATCATGGACAAGATCA | qRT-PCR |
|  |  | CAAACTCACACAGCACACAC |  |
| *PvC3H72* | Pavir.J07041.1 | TGGGCTTCTACCTGGATCTC | qRT-PCR |
|  |  | CATCAGCCTGTGCATCTCTT |  |
| *PvFTSH4* | _ | TGGATGGCTTTAAGCAGAATGA | qRT-PCR reference gene |
|  |  | CAAAACGCCCAGGTCTGACT |  |
| *HTPII* | _ | CAAACTGTGATGGACGACACCG | PCR/Southern blot |
|  |  | TATATGCTCAACACATGAGCG |  |
| *PvC3H72* | Pavir.J07041.1 | ttgt gaattc ATGGGCGACCTTGCTGATC | PCR |
|  |  | tataagcttTTTCGGCTCCAAGTGCATCTG |  |

**Table S2 Primers used in this study**
